# Supplementary material for: Effects of experimental situation on group cooperation and individual performance: Comparing laboratory and online experiments
Source: PLoS One. 2022 Apr 20;17(4):e0267251. doi: 10.1371/journal.pone.0267251 (PMC9020741; doi:10.1371/journal.pone.0267251)
Supplement: S1 File — Supplementary analysis including S1–S7 Tables and S1–S3 Figs, and supplementary method. (DOCX) [file pone.0267251.s001.docx]

Supporting Information for

Effects of experimental situation on group cooperation and individual performance: comparing laboratory and online experiments

Hiroki Ozono and Daisuke Nakama

Hiroki Ozono

Email: hiroki.ozono@gmail.com

**This PDF file includes:**

Supplementary analysis (including S1-S7 Tables and S1-S3 Figs)

Supplementary method

**Other supplementary materials for this manuscript include the following:**

Supplementary data

**Supplementary analysis**

1. **Analysis of the demographic factors and characteristics per sample group**

**S1 Table. The demographic factors and characteristics per sample group**

**Note: F, M and N for gender indicate Female, Male and N/A, respectively. P, I, C and A for SVO (Social Value Orientation) indicate Prosocial, Individualistic, Competitive and Altruistic, respectively.**

**Fisher’s exact tests were conducted for gender and SVO.**

The participants in the laboratory and online experiments were randomly assigned, and no significant differences in demographic factors or personality were found.

There were significant differences between students and YCrowd participants in terms of gender and age. However, the multiple regression analysis in S2-S7 Tables showed that, after controlling for these demographic factors, the trends did not fundamentally change from those reported in the main text.

**２．Multiple regression analyses in group task.**

**S2 Table. PGG contribution with no-punishment**

**Note: we constructed multilevel regression models that considered random intercepts for each group and individual when the coefficients were estimated. “Period” is period number, “Lab student” is a dummy for the Lab student sample, “Online-YCrowd” is a dummy for the YCrowd sample, and “Gender” is a dummy for the gender (Female = 0, Male = 1). Standard errors are in parentheses and p-values in square brackets.**

*** p < 0.1; ** p < 0.05; *** p < 0.01**

**S3 Table. PGG contribution with punishment**

 **Note: we constructed multilevel regression models that considered random intercepts for each group and individual when the coefficients were estimated. “Period” is period number, “Lab student” is a dummy for the Lab student sample, “Online-YCrowd” is a dummy for the YCrowd sample, and “Gender” is a dummy for the gender (Female = 0, Male = 1). Standard errors are in parentheses and p-values in square brackets.**

*** p < 0.1; ** p < 0.05; *** p < 0.01**

**S4 Table. Punishment received in PGG**

**Note: we constructed multilevel regression models that considered random intercepts for each group and individual when the coefficients were estimated. “Period” is period number, “Lab student” is a dummy for the Lab student sample, “Online-YCrowd” is a dummy for the YCrowd sample, and “Gender” is a dummy for the gender (Female = 0, Male = 1). Standard errors are in parentheses and p-values in square brackets.**

*** p < 0.1; ** p < 0.05; *** p < 0.01**

Similar to the trend reported in the main text, there was no significant difference in the PGG contribution between the laboratory and online, and significant difference between students and YCrowd participants. There were no differences in punishment received among conditions.

The only differences from the results reported in the main text were the non-significance of PGG contribution with and without punishment condition between student and YCrowd samples after controlling for age and gender. This might be due to the strong relationship between age and sample heterogeneity. As seen in Analysis 1 (S1 Table), age was very different between the student and YCrowd samples, so it was difficult to distinguish the effects of sample heterogeneity and of age.

To discuss in detail, we show the PGG contribution with and without punishment condition by age group.

**S1 Fig. The PGG contribution without punishment condition by age group.**

**Note: Error bars indicate standard errors.**

**S2 Fig. The PGG contribution with punishment condition by age group.**

**Note: Error bars indicate standard errors.**

In the YCrowd sample, there was no linear relationship between age and contribution. Therefore, it is difficult to interpret whether the student sample contributed more because of their younger age. We interpret that other differences in the sample groups, rather than age, explain the difference in the amount of contribution.

**3．The regression analyses for individual tasks.**

**S5 Table. The regression analysis of anagram task**

**Note: We constructed regression analysis with individuals. “Order of the tasks” is the order of three tasks, “lab student” is a dummy for the lab student sample, “online-YCrowd” is a dummy for the YCrowd sample, and “Gender” is a dummy for the gender (Female = 0, Male = 1). Standard errors are in parentheses and p-values are in square brackets.**

*** p < 0.1; ** p < 0.05; *** p < 0.01**

**S6 Table. The regression analysis of inverse-anagram task**

**Note: We constructed regression analysis with individuals. “Order of the tasks” is the order of three tasks, “lab student” is a dummy for the lab student sample, “online-YCrowd” is a dummy for the YCrowd sample, and “Gender” is a dummy for the gender (Female = 0, Male = 1). Standard errors are in parentheses and p-values are in square brackets.**

*** p < 0.1; ** p < 0.05; *** p < 0.01**

**S7 Table. The regression analysis of remote association task**

**Note: We constructed regression analysis with individuals. “Order of the tasks” is the order of three tasks,** **“lab student” is a dummy for the lab student sample, “online-YCrowd” is a dummy for the YCrowd sample, and “Gender” is a dummy for the gender (Female = 0, Male = 1). Standard errors are in parentheses and p-values are in square brackets.**

*** p < 0.1; ** p < 0.05; *** p < 0.01**

We found a similar tendency to that reported in the main text, with only one result differing from that in the main text. In the in-anagram task, the YCrowd participants performed better than the students although we did not find such a tendency with the ANOVA in the main text. The factor of age affected the results – older participants performed less well than younger participants in YCrowd sample and, after controlling for this, we found better performance by YCrowd participants.

We can interpret this tendency as follows: YCrowd participants are more accustomed to do simple and dull tasks such as the in-anagram because crowd-sourcing services demand such types of work.

However, we need to consider this more carefully. This might be due to the strong relationship between age and sample heterogeneity as discussed in the above section. The age was very different between student and YCrowd sample, so it was difficult to distinguish the effects of sample heterogeneity and of age. To discuss in detail, we show the number of correct responses for in-anagram by age group.

**S3 Fig. The number of correct responses of inverse-anagram task by age group**

**Note: Error bars indicate standard errors.**

In the YCrowd sample, there was a negative linear relationship between age and performance for the in-anagram. Therefore, it is possible to interpret that the YCrowd sample performed the in-anagram task better. However, performances of those aged in their 30s, 40s and 50s, who were the majority of the YCrowd sample (89%), did not differ much from that of the student sample, so there seems no need to be concerned in this regard.

**Supplementary method**

*In the main experiment for the student sample, after a brief verbal introduction, participants read the following instructions on the computer monitor telling them that they would take part in an experiment on decision making.*

*In the additional experiment for the general sample, participants directly read the instructions.*

*The instructions below are based on the main experiment. When the instructions differed in the additional experiment, they are written in parentheses.*

1. **General guidelines**

**Introduction**

Duration of the experiment:

- It will take about 80 minutes (in the additional study, 40 minutes) to complete the entire experiment. If you cancel your participation in the middle of the experiment, we will not be able to pay you the additional rewards.
- If you have other things to do during the experiment, please cancel your participation now and come back when you have more time.

Notes:

- Do not use the “back” button in the browser. The experiment may not proceed properly and you may not receive the reward.
- The “Refresh” button can be used when the screen freezes, but please do not unnecessarily use this button.

Handling of personal information in the experiment:

- The experiment will be conducted anonymously.
- All data will be stored and processed in an anonymous manner.
- Please be assured that your personal information will not be leaked outside the experiment.
- The experimental results will be used for educational and research purposes only and will not be used for any other purpose.

Please click the “Next” button below to start the experiment only if you are satisfied with and agree to all the points explained above.

1. **Instructions for group tasks**

*In the main experiment for the student sample, the participants performed both group tasks and individual tasks in this order. In the additional experiment for the general sample, the participants performed either group tasks or individual tasks.*

**2.1. Instructions for PGG without punishment**

**Explanation of transactions**

Formation of groups:

Three-person groups will be randomly formed from the participants in the experiment. In the three-person groups, participants will repeat transactions 10 times. The members of the three-person groups will not be changed during the experiment. Please note that the number of points earned from the transactions will directly reflect the actual amount of your reward. Details of the reward will be explained at the end of instructions.

We will now explain the transactions in detail, followed by a confirmation test. You will not be able to proceed to the transactions until you have answered all the questions correctly, so please read the following explanation carefully.

About transactions:

At the beginning of each transaction, each participant will be given 20 points.

Each participant decides how much of the 20 points to provide to the group, in units of 1 point.

For example, if you provide 5 points to the group, you will have 20 points − 5 points = 15 points at that moment.

Input screen for provision:

The following is a screenshot of the actual input screen for determining the amount to provide. Please make sure you know how to use it.


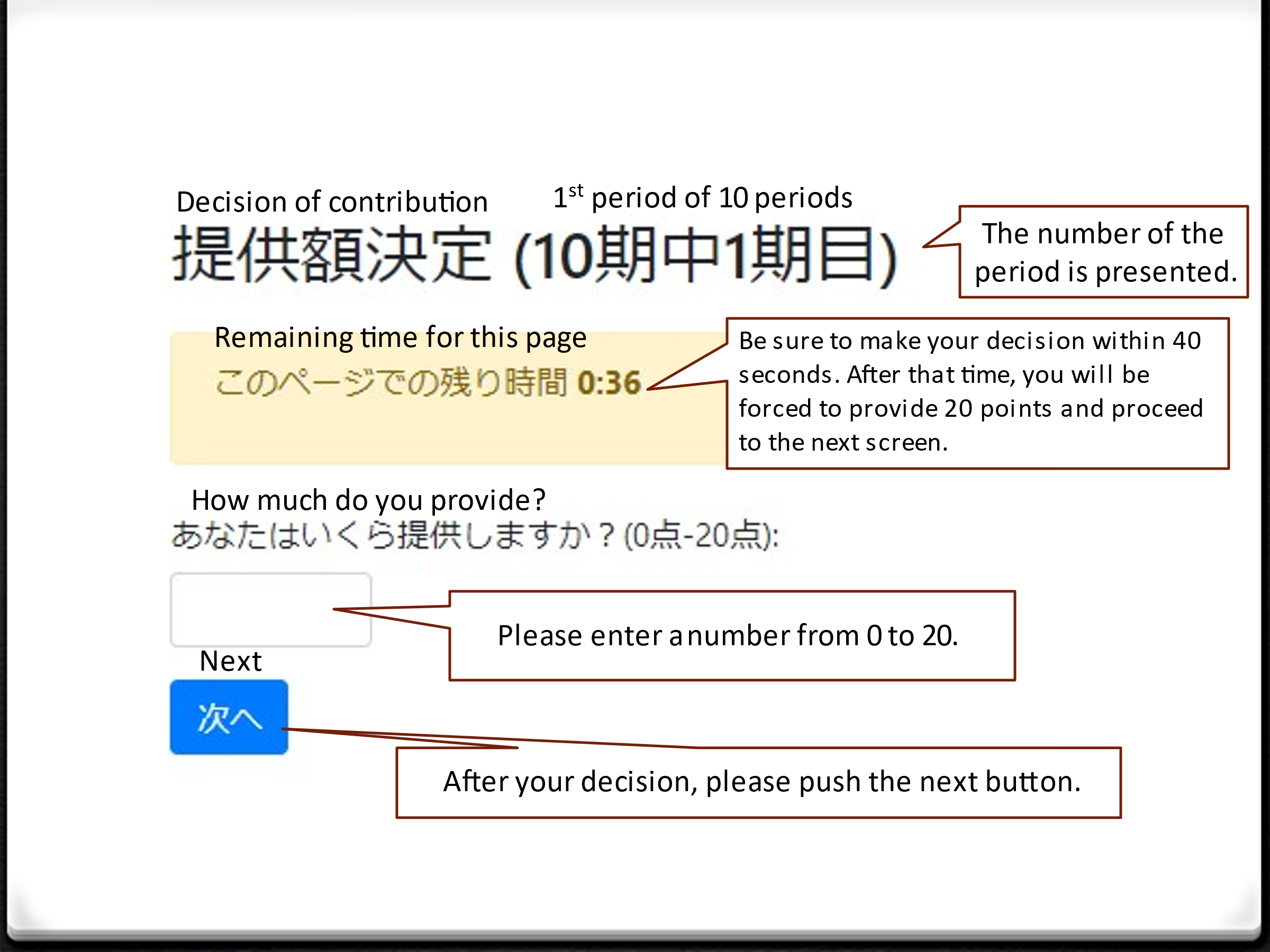


Distribution of the total provided amount:

After everyone has decided on the amount to provide, the distribution amounts to all participants are determined according to the following rule.

The rule is: “For every 1 point given to the group, 0.5 points are given to all participants.” For example, if the total amount provided by the three people in the group is 30 points, then 30 points × 0.5 = 15 points will be distributed to each of the three people.

In addition, participants will be informed how much each of the three people provided after all members have made their decisions.

Summary so far:

The first step is to decide how much of the 20 points you will provide to the group. At that moment, you will be left with 20 points − (the points you offered).

After that, 0.5 point per point provided by a participant is distributed to each participant depending on how many points three members in the group have provided. In other words, the points you earn will be as follows:

[20 points – (your provision)] + [(total of the provision of all three players) × 0.5 points]

Next, we will explain with examples to help you better understand.

Example 1:

For example, if all three members provide 20 points (20 points × 3 members = 60 points total), each member will earn 30 points [(20 – 20) + (60 × 0.5) = 30].

If all three members do not provide any points, then each member will earn 20 points [(20 − 0) + (0 × 0.5) = 20].

In this way, the group as a whole will be more profitable when everyone provides their points than when everyone does not.

Example 2:

Next, consider the case where you do not provide any points at all, but both of the other two people provide 20 points (20 points × 2 people = 40 points total).

In this case, you will earn 40 points [(20 − 0) + (40 × 0.5) = 40].

Each of the other two members will earn 20 points.

In this way, you will earn more points when you do not provide your points to the group than when you provide them.

Example 3:

So far, you learned with extreme examples, such as “providing all 20 points,” but in reality, the decision will be made on a per-point basis, so calculation will be a bit more complicated.

For example, if you provide 15 points and the other members provide 25 points in total, the total amount provided by the three members will be 40 points (15 points + 25 points).

Therefore, your earnings will be 25 points [(20 – 15) + (40 × 0.5) = 25]

Result screen:

Below is an example of the results screen that appears at the end of each transaction. Please make sure you understand how the results are displayed (the actual points will vary depending on your choices).

First, the amount of each member’s provision will be shown.


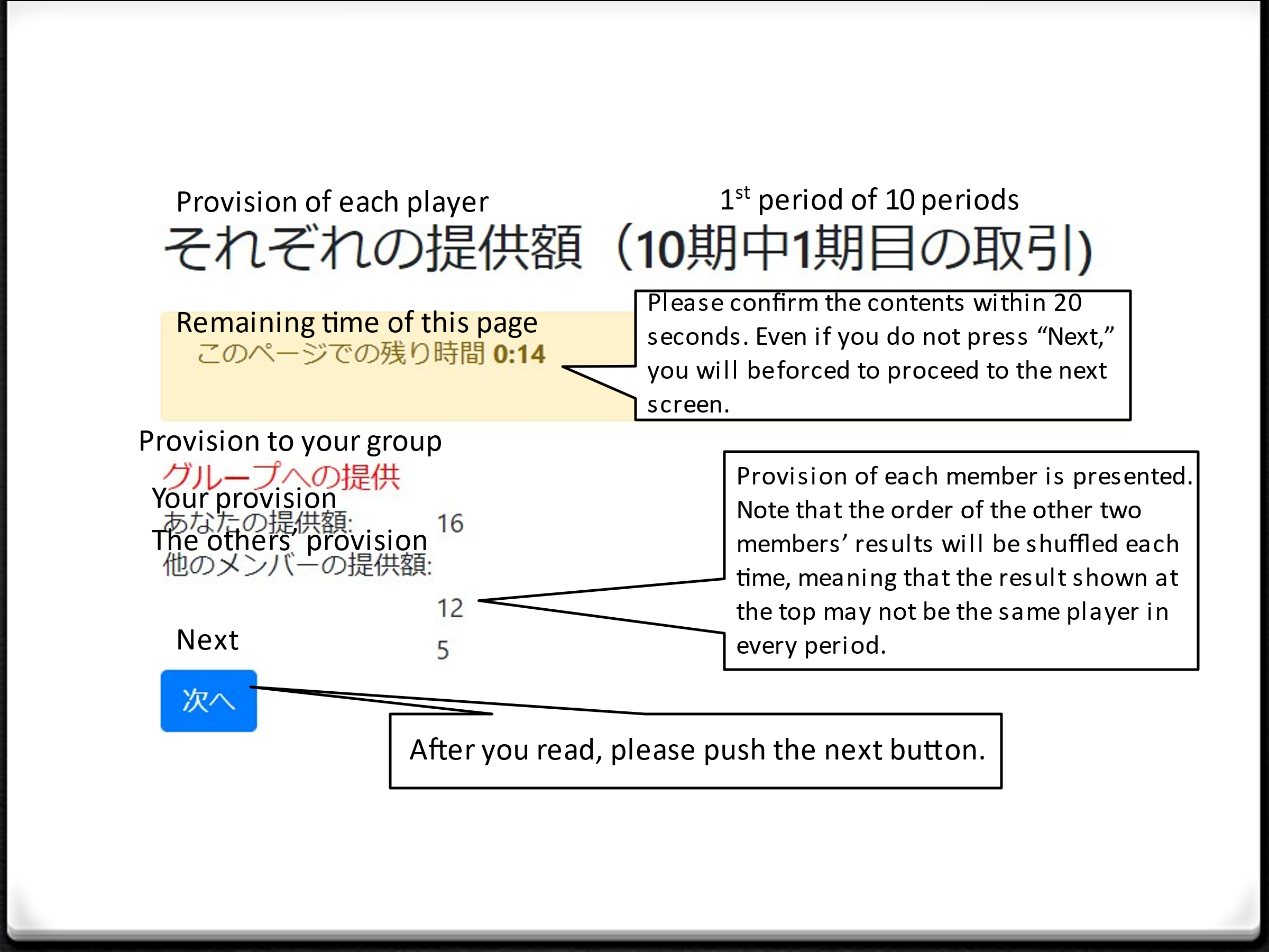


Next, how much you have earned for that transaction will be shown.


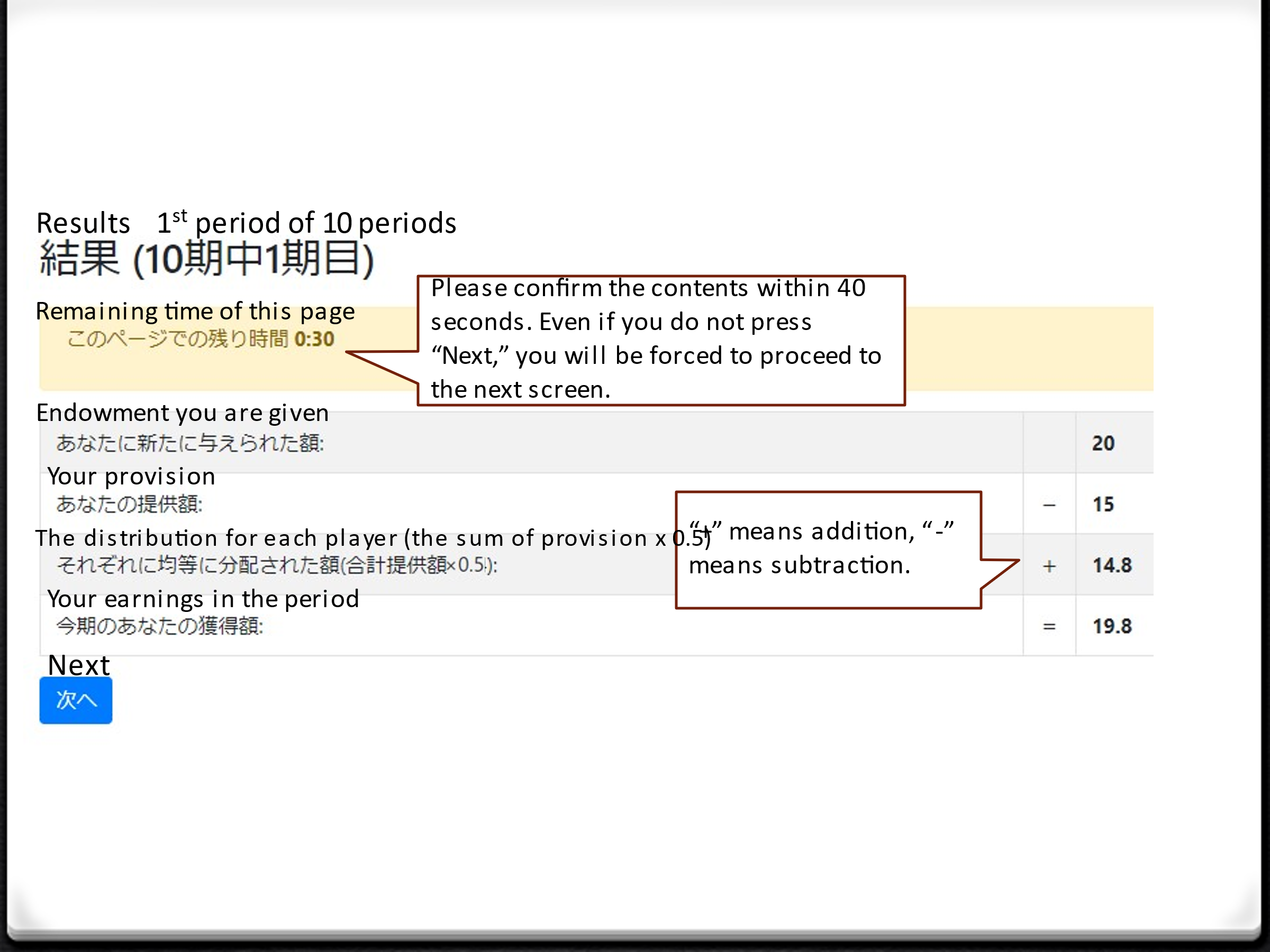


Summary:

The process of a single transaction is as follows:

“Provision to the group (how much of the 20 points will you offer)”

↓

“Calculation of the points earned”

This transaction will be repeated 10 times with the same members.

About rewards:

The total of the points you earned in 10 transactions will be the additional rewards. Specifically, 1 point in the experiment will be converted into 1 yen (in the additional study, 0.2 yen).

As mentioned above, the results of the transaction will be directly reflected in your rewards, so please make serious decisions in every transaction.

**Confirmation test**

1. Do the three members of the group stay the same? Do they change with each period?

A: stay the same

B: change with each period

(Answer: A)

2. In one period, your provision was 20 points. The other two players also provided 20 points. In this case, how many points do you get?

A: 10

B: 20

C: 30

(Answer: C)

3. In one period, your provision was 0 points. The other two players also provided 0 points. In this case, how many points do you get?

A: 0

B: 10

C: 20

(Answer: B)

4. In one period, your provision was 0 points. The other two players provided 20 points. In this case, how many points do you get?

A: 20

B: 30

C: 40

(Answer: C)

5. In one period, your provision was 10 points. The other two players provided 10 points. In this case, how many points do you get?

A: 15

B: 20

C: 25

(Answer: C)

**2.2. Instructions for punishment**

*The following instructions are presented after the participants completed the PGG without punishment.*

Introduction for the reduction stage:

This is the same as the earlier transaction up to the point where all members determined their providing amounts. After that, there is a reduction stage. The reduction stage is explained in detail below.

Explanation for the reduction stage:

In the reduction stage you can see how much other members have provided to you.

You can use your earnings to reduce other members’ earnings.

Specifically, if you use 1 point to reduce a particular member, you lose that 1 point, but your opponent loses 2 points.

For example, if you use 2 points for a reduction to person B, their earnings will be reduced by 4 points.

You can use a maximum of 10 points for the reduction. Of course, you do not have to use even 1 point. Below is an example of the actual decision screen:


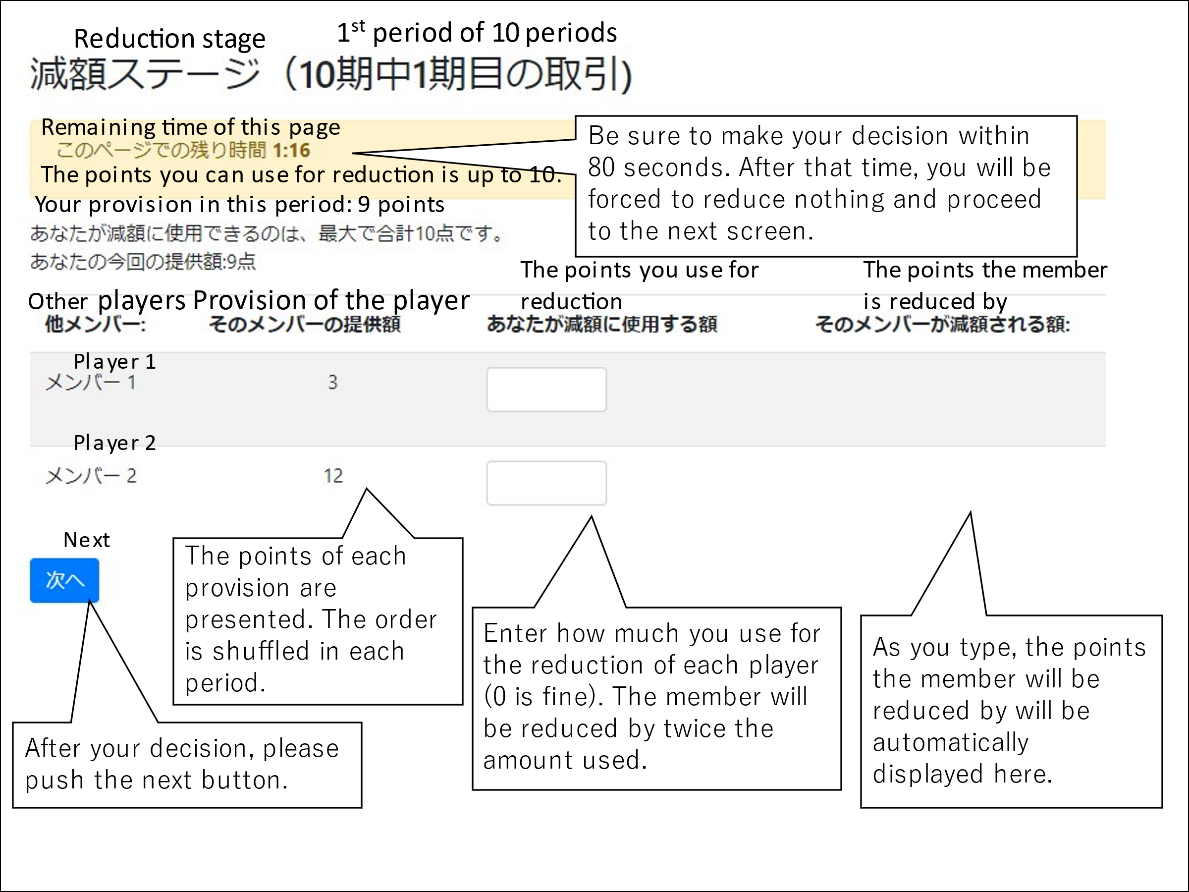


Next, how much you have earned for that transaction will be shown.


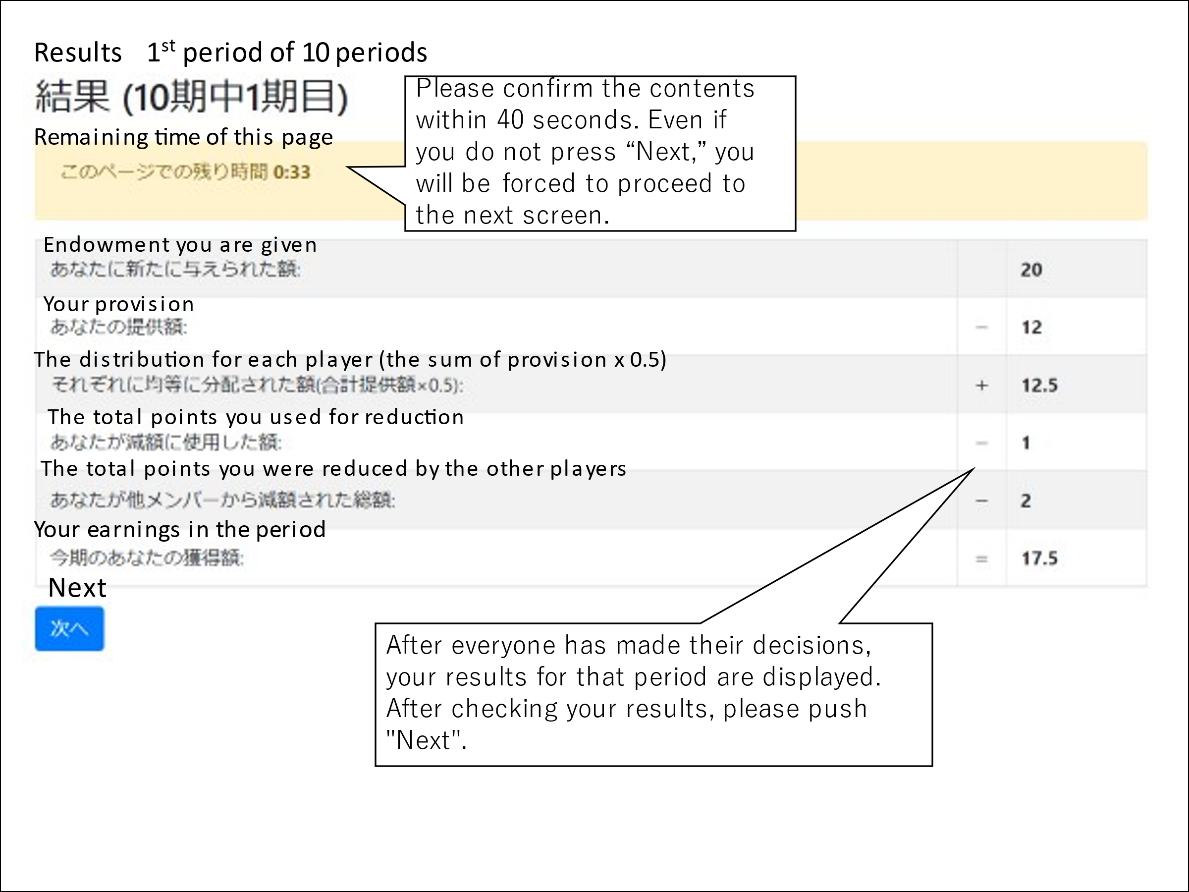


**Confirmation test**

1. Suppose you have spent a total of 2 points to reduce the other members and the other members have spent a total of 3 points to reduce you. How much will you lose in the reduction stage?

A: 3

B: 5

C: 8

(Answer: C)

**3. Instructions for individual tasks**

*The order of three individual tasks was partly counterbalanced. In the main study, the order of the three tasks was either anagram–in-anagram–remote association task or in-anagram–anagram–remote association task. Since anagram and in-anagram tasks were similar for the participants, we regarded them as one unit to simplify the instruction process and to enhance the smooth transition between tasks. In the additional study, half of the participants did the remote association task first and the other half did it last. The order of anagram and in-anagram task was also counterbalanced. There were no statistical differences due to the order of the tasks.*

**3.1. Instructions for anagram task**


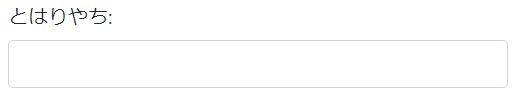
You will be presented with a five-letter word as shown below.

*Note: “とはりやち” does not have any meaning like “REOHS”*

Rearrange them to make a meaningful word (in the example above, the correct answer is “はやとちり”).


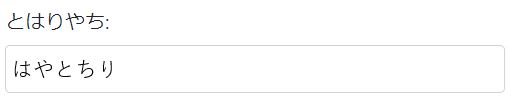
Type your answer in hiragana in the box below.

*Note: “はやとちり” has a meaning like “HORSE”*

In the experimental session, about 18 similar questions will be presented at the same time, so please try your best to answer as many questions as possible within the time limit of 4 minutes.

The order of solving the problems does not have to be from the top to the bottom. You can start with the questions that you can answer.

When the time limit of 4 minutes has expired, the screen will automatically switch to another 18 questions, and you will answer the new problems in the same way.

You will repeat this 2 times. In other words, you will consecutively work on this task for a total of 8 minutes (4 minutes × 2 times).

<For Contingent-pay condition>

Note that you will receive an additional reward of 8 yen (in the additional study, 3 yen) for each correct answer. The more questions you answer correctly, the more reward you will receive. Please try to answer as many questions as possible.

<For Fixed-pay condition>

Note that you will receive the fixed amount of 100 yen (in the additional study, 35 yen for additional reward regardless of the number of correct answers). Even though correct answers will not increase the rewards, please try to obtain as many correct answers as possible.

**3.2. Instruction for inverse-anagram task**


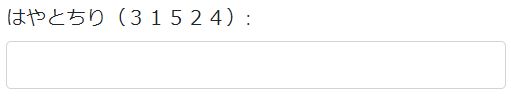
You will be presented with a five-letter word as shown below.

*Note: “はやとちり” has a meaning like “HORSE”*


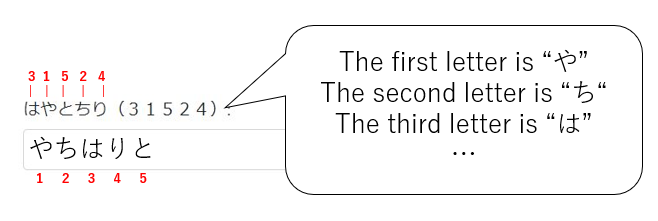
Rearrange the five letters in the same order as the numbers indicate (in this example, the first letter is “や,” the second letter is “ち,” and so on, and the correct answer is “や　ち　は　り　と”).

*Note: “とはりやち” does not have any meaning like “REOHS”*

In the experimental session, about 50 similar questions will be presented at the same time, so please try your best to answer as many questions as possible within the time limit of 4 minutes.

The order of solving the problems does not have to be from the top to the bottom. You can start with the questions that you can answer.

When the time limit of 4 minutes has expired, the screen will automatically switch to another 50 questions, and you will answer the new problems in the same way.

You will repeat this 2 times. In other words, you will consecutively work on this task for a total of 8 minutes (4 minutes × 2 times).

<For Bonus condition>

Note that you will receive an additional reward of 5 yen (in the additional study, 1 yen) for each correct answer. The more questions you answer correctly, the more reward you will receive. Please try to answer as many questions as possible.

<For Fixed-pay condition>

Note that you will receive the fixed amount of 100 yen (in the additional study, 35 yen) for additional reward regardless of the number of correct answers. Even though correct answers will not increase the reward, please try to obtain as many correct answers as possible.

**3.3. Instruction for remote association task**


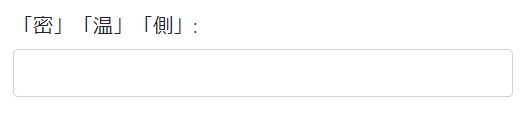
You will be presented with three kanji as shown below.

*Note: These are three different kanji characters (Japanese ideographs based on Chinese characters).*

*Think of the kanji that is commonly followed by these three kanji (in this example, the correct answer is “室” (密室、温室、側室).*


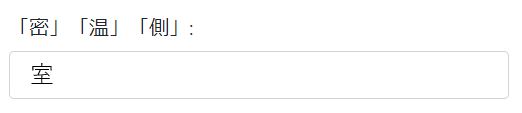
Please enter your answer in kanji characters in the box below.

You will be presented with about 20 similar questions at the same time, so please try your best to answer as many as possible within 4 minutes.

The order of solving the problems does not necessarily have to be from the top to the bottom, and you can start with the problems you can answer.

After the time limit of 4 minutes has expired, the screen will automatically switch to another 20 questions, and you will answer them in the same way.

There will be a total of 2 repetitions. In other words, you will consecutively work on this task for a total of 8 minutes (4 minutes × 2 times).

<For Bonus condition>

Note that you will receive an additional reward of 15 yen (in the additional study, 5 yen) for each correct answer. The more questions you answer correctly, the more reward you will receive. Please try to answer as many questions as possible.

<For Fixed-pay condition>

Note that you will receive the fixed amount of 100 yen (in the additional study, 35 yen) for additional reward regardless of the number of correct answers. Even though correct answers will not increase the reward, please try to obtain as many correct answers as possible.

**3.4. Practice sessions (same for the three tasks)**

*After each instruction, the participants were given two trial questions to answer with a time limit of one minute. If they got them wrong, they were told the correct answers and asked to answer the same questions again for confirmation.*

**3.5. Instructions in task (same for the three tasks)**

*After the practice session, the participants were shown several questions and asked to answer as many questions as possible. They were prohibited from searching for the answers on the web.*

*Please see the questions in each task in the supplemental data.*

**4. Post experimental questionnaire**

*Please see the items of the post questionnaire in the supplemental data.*
