# Supplementary material for: Effects of experimental situation on group cooperation and individual performance: Comparing laboratory and online experiments
Source: PLoS One. 2022 Apr 20;17(4):e0267251. doi: 10.1371/journal.pone.0267251 (PMC9020741; doi:10.1371/journal.pone.0267251)
Supplement: Attachment — Submitted filename: Review_PONE-D-21-25066.pdf [file pone.0267251.s003.pdf]

Effects of experimental situation on group cooperation and individual performance: comparing laboratory and online experiments

The paper examined how experimental situation, more precisely, online or lab setting affects group cooperation and other individual tasks that measure performance.

In the first experiment, the author assigned students randomly to one of the treatments. In this experiment, it is plausible to measure the effect of online setting compared to lab setting.

Also, an online experiment using Yahoo crowd was made (Ycrowd). But this experiment involves another type of sample (with different characteristics).

Results from experiment 1 suggest no difference in cooperation, while comparing Ycrowd with Lab setting the results show differences.

General comments:

1. The random assignment makes the two treatments (online or lab setting) comparable, and suggest internal validity. This assures that author could find the causal effect of the treatment.
2. However, in experiment Ycrowd this is not truth. These subjects, probably are not comparable, and even if they were, the non-observable characteristics of this group would be different compare to lab students. Saying this, It is not clear for me how this experiment would add more information about the topic (which is very interesting for me and I agree with the author that this kind of research is necessary in the literature). I suggest the following comments:

- First, author need to show that in experiment 1 both treatments were balance in different observable characteristics (mean tests and p values shown in a table).
- For the second comment, author need to show that both samples (Ycrowd and Lab) are comparable. A paper that analyzed this is Brañas-Garza et al. (2013) "Experimental subjects are not different", I suggest to add it in the introduction.
- Third, the null effects in the first experiment could be explained it because a lack of statistical power calculations. I suggest to run power calculation in other to see if it is possible to have significant results with a higher number of observations.
- Fourth, author need to add tables with regression results controlling for age, gender and others controls.

Specific comments:

1. The English needs to be revised:
    a. For example, in the first phrase (line 25) appears "perform something", which sounds wear.
    b. Also lines 31 to 333.
    c. What does "behavioral tendencies" means? I thinks it refers to standard behaviour or even could be results (line 33).
    d. A more appropriate terms is heterogenous sample than general sample in line 31. Also could be non-standard experimental subjects.
    e. Line 37, again general sample online. Also it is not the effect of the population, it is the effect of the sample heterogeneity.

f. I would not use the term population, because these type of subjects (Mturkers or others) are not representative of the whole population.
g. Line 8: there is a typo is Arechar.

2. The structure does not follows the guideline instructions. It is too long and needs to follows the structure of Plos One´s papers.
