# Supplementary material for: Effects of experimental situation on group cooperation and individual performance: Comparing laboratory and online experiments
Source: PLoS One. 2022 Apr 20;17(4):e0267251. doi: 10.1371/journal.pone.0267251 (PMC9020741; doi:10.1371/journal.pone.0267251)
Supplement: Attachment — Submitted filename: Reply letter _final.docx [file pone.0267251.s005.docx]

March 29, 2022

Dear Dr. Alfonso Rosa Garcia

Thank you again for providing my colleagues and I with the opportunity to revise and resubmit our manuscript (PONE-D-21-25066) to *PLOS ONE*. We appreciate the feedback that you and the reviewers provided and have revised our manuscript accordingly. We believe that we have clearly and thoroughly addressed each of the issues raised by you and the reviewers, and think that our paper has been strengthened considerably by the changes that we have made. Below we provide the decision letter and reviews, with our responses (marked with asterisks) below each point. Please feel free to contact me if you have any questions about our resubmission.

Thank you again. We look forward to hearing from you.

Sincerely,

Hiroki Ozono

========================

**We greatly appreciate the comments and careful review of our paper by the editor and two reviewers. We have revised the paper taking into account the reviewers’ helpful suggestions and comments. We now address each comment made by the editor and two reviewers. Our responses are marked with asterisks.*

**[Reply to the Editor]**

**Editor’s Comment:**

Dear Dr. Ozono,

Thank you for submitting your manuscript to PLOS ONE. After careful consideration, we feel that it has merit but does not fully meet PLOS ONE’s publication criteria as it currently stands. Therefore, we invite you to submit a revised version of the manuscript that addresses the points raised during the review process.

I consider that your paper address now the previous concerns but, as one referee suggests, there are some considerations that could improve the presentation of the findings. So, please consider how to include the suggestions done by Reviewer 3. They are minor changes that could be easily used to improve the paper.

Please submit your revised manuscript by Apr 14 2022 11:59PM. If you will need more time than this to complete your revisions, please reply to this message or contact the journal office at plosone@plos.org. When you're ready to submit your revision, log on to https://www.editorialmanager.com/pone/ and select the 'Submissions Needing Revision' folder to locate your manuscript file.

Please include the following items when submitting your revised manuscript:

A rebuttal letter that responds to each point raised by the academic editor and reviewer(s). You should upload this letter as a separate file labeled 'Response to Reviewers'.

A marked-up copy of your manuscript that highlights changes made to the original version. You should upload this as a separate file labeled 'Revised Manuscript with Track Changes'.

An unmarked version of your revised paper without tracked changes. You should upload this as a separate file labeled 'Manuscript'.

If you would like to make changes to your financial disclosure, please include your updated statement in your cover letter. Guidelines for resubmitting your figure files are available below the reviewer comments at the end of this letter.

If applicable, we recommend that you deposit your laboratory protocols in protocols.io to enhance the reproducibility of your results. Protocols.io assigns your protocol its own identifier (DOI) so that it can be cited independently in the future. For instructions see: https://journals.plos.org/plosone/s/submission-guidelines#loc-laboratory-protocols. Additionally, PLOS ONE offers an option for publishing peer-reviewed Lab Protocol articles, which describe protocols hosted on protocols.io. Read more information on sharing protocols at https://plos.org/protocols?utm_medium=editorial-email&utm_source=authorletters&utm_campaign=protocols.

We look forward to receiving your revised manuscript.

Kind regards,

Alfonso Rosa Garcia

Academic Editor

PLOS ONE

**Reply:**

**We thank you for giving us the opportunity to revise our manuscript. We made a point-by-point response to all issues raised by the reviewers.*

**[Reply to the Journal Requirements:]**

Journal Requirements:

Please review your reference list to ensure that it is complete and correct. If you have cited papers that have been retracted, please include the rationale for doing so in the manuscript text, or remove these references and replace them with relevant current references. Any changes to the reference list should be mentioned in the rebuttal letter that accompanies your revised manuscript. If you need to cite a retracted article, indicate the article’s retracted status in the References list and also include a citation and full reference for the retraction notice.

**Reply:**

** We appreciate your suggestion. We do not refer to retracted papers.*

**[Reply to the Reviewer 1]**

**Reviewer 1’s Comment (1):**

You have addressed the comments made in a well-made fashion, therefore I accept the paper. I thank you for quoting our paper in the new version of this paper.

**Reply:**

** We appreciate the important comments by the reviewer concerning the first version of our paper.*

**[Reply to the Reviewer 3]**

**Reviewer 3’s Comment (1):**

Reviewer #3: The authors have addressed the reviewers’ comments adequately, in my opinion. This piece offers new interesting insights to a growing literature. Thus, the 11 comments below concentrate on the presentation of the findings, rather than on their fundamentals. I think such comments can be addressed relatively quickly, if the authors find them useful.

1. I believe the readership could assimilate your design more easily in the context of an explicit 2x2x2 environment [experimental situation x incentive scheme x sample heterogeneity] where you have the first four combinations covered [experimental situation x incentive scheme] in the first two research questions. Then, you could present a case for the third set of combinations [sample heterogeneity], where you focus only on online situations – e.g. it is fine to have the most elusive combinations [lab & heterogeneous] outside the scope of this type of paper because of the logistical and practical insights obtained. This approach would also allow you to integrate the “Additional Experiment” section more organically, potentially as a third research question.

**Reply:**

*** *We appreciate your suggestion. Indeed, this research frame (2×2×2) would be more integrative. However, we did not have such a 2×2×2 frame at the start of the study, and the online-worker sample was actually collected "additionally". In recent years, researchers have argued that the research frame should be reported as it is without changing it a posteriori (Editorial, 2020, Nature Human Behavior), and we would like to follow this policy. Therefore, we decided to just mention that we did not obtain data for the online-worker sample with the laboratory situation and the reasons for this in the Introduction (page 10 line 159 to 163).*

*.*

**Reviewer 3’s Comment (2):**

2. Page 5, line 66. It is not clear to me why social distance theory predicts substantially different (prosocial) behavior. The way it is explained seems quite in line with the idea suggested by the observer effect (i.e. that prosociality would flourish with physical proximity, including the experimenter).

**Reply:**

*** *Indeed, these two theories are conceptually similar. In this study, we do not focus specifically on the differences between the two theories, but simply trace the discussions in previous studies that compared the laboratory situation with the online situation. We added sentences to clarify our thought**s (page 5 line 70 to 73).*

**Reviewer 3’s Comment (3):**

3. Page 6, line 79. Add “, that participants online tend to be less prosocial”.

**Reply:**

** We appreciate your suggestion. We added the sentence (page 6 line 82).*

**Reviewer 3’s Comment (4):**

4. Page 6 line 84. I’d argue that it also warrants a more nuanced comparison of the results because the papers discussed, although focused on experimental situations, offer insights based on samples from different countries. Therefore, to the extent that there is an interaction between situations and sample compositions, pooling their results is not ideal.

**Reply:**

*** *We appreciate your suggestion. As you stated, these inconsistent results might be due to differences in sample populations, and it might not be appropriate to simply pool them together. However, all the experiments we referred to were conducted with university students in Western countries, and even in the same country (Germany), with inconsistent results (Bader et al., 2019; Schmelz & Ziegelmever, 2020). It is difficult to obtain other detailed information regarding the sample groups, so we cannot discuss this point further. We added this argument in the Introduction (page 6 line 87 to 92).*

**Reviewer 3’s Comment (5):**

5. Page 7 line 91. I believe they found the online sample, which was older, to be more cooperative – even after controlling for demographics / overall sample heterogeneity.

**Reply:**

** Indeed, they found that older participants were more cooperative even after controlling for other demographic factors. However, they found this in the no-punishment condition (see Table 6 of Arechor et al., 2018) and we do not think it is necessary to refer to it in this context.*

**Reviewer 3’s Comment (6):**

6. Research question 2. This question could emphasize a bit more that the comparisons rely on student samples. Ideally, you could also highlight that the hypotheses relative to performance are quite context-dependent: it is very different to compare a sample of students in the lab and online versus, for example, crowds that opt to take surveys out of boredom while commuting or another group that takes surveys for a living and concentrates in front of their computers.

**Reply:**

*** *We appreciate your suggestion. We added the sentences in the limitation (page 39 line 685 to 688).*

**Reviewer 3’s Comment (7):**

7. Page 10 line 156. This argument could be strengthened by citing Fehr & Gächter (2002), who found no evidence of order effects.

**Reply:**

** We appreciate your advice. We added the reference to this study (page 11 line 172 to 173).*

**Reviewer 3’s Comment (8):**

8. Page 11 line 179. I think this line should contextualize the prevalence of a pandemic and the potential effect on who decides to go to a lab.

**Reply:**

** We agree with you. We added sentences discussing the potential selection bias (page 12 line 194 to 197).*

**Reviewer 3’s Comment (9):**

9. Page 17 line 286. It would be informative to provide brief statistics about how many groups were excluded here, and how many participants timed out (none?). A line justifying why full contributions were set as the default (and the inclusion of an automatic program) would be useful too.

**Reply:**

** There was no timeout in the experiment and we mentioned this in the beginning of the results. For better understanding, we added a sentence also in this paragraph (page 18 line 306 to 307). In regard to the second point, we explained why the full contribution was set as the default (page 18 line 302 to 304).*

**Reviewer 3’s Comment (10):**

10. Page 19 line 309. It would be useful to know more about such a preliminary experiment.

**Reply:**

** We added the information about the preliminary experiment (page 20 line 329 to 331).*

**Reviewer 3’s Comment (11):**

11. Page 21 line 345. Results from the Yahoo crowdsourcing sample currently come a bit out of the blue. I think this sample should be contextualized a bit more before it is introduced here (e.g. by integrating the study as suggested in point 1), and in the Figures. Other than the abstract, no mention of YCrowd is made until this point.

**Reply:**

** We appreciate your suggestion. We briefly explained the YCrowd in the Introduction section (page 10 line 154 to 156).*

**References in this reply letter**

*Editorial. Tell it like it is. Nature Human Behaviour, 2020; 4 (1), 1.*

*Bader F, Baumeister B, Berger R, Keuschnigg M. On the transportability of laboratory results. Sociol Methods Res. 2019; 0049124119826151.*

*Schmelz K, Ziegelmeyer A. Reactions to (the absence of) control and workplace arrangements: experimental evidence from the internet and the laboratory. Exp Econ. 2020; 23(4): 933-60.*

*Arechar AA, Gächter S, Molleman L. Conducting interactive experiments online. Exp Eco. 2018; 21(1): 99-131.*

*Fehr E, Gächter S. Altruistic punishment in humans. Nature. 2002; 415(6868), 137-140.*
